# Interpreting the spectrum of gamma-secretase complex missense variation in the context of hidradenitis suppurativa—An *in-silico* study

**DOI:** 10.3389/fgene.2022.962449

**Published:** 2022-09-02

**Authors:** Dillon Mintoff, Nikolai P. Pace, Isabella Borg

**Affiliations:** ^1^ Department of Pathology, Faculty of Medicine and Surgery, University of Malta, Msida, Malta; ^2^ Centre for Molecular Medicine and Biobanking, University of Malta, Msida, Malta; ^3^ Centre for Molecular Biology and Biobanking, University of Malta, Msida, Malta; ^4^ Department of Anatomy, Faculty of Medicine and Surgery, University of Malta, Msida, Malta; ^5^ Department of Pathology, Mater Dei Hospital, Msida, Malta

**Keywords:** hidradenitis suppurativa, genetics, gamma secretase complex, pathophysiology, nicastrin

## Abstract

Hidradenitis suppurativa (HS) is a disease of the pilosebaceous unit characterized by recurrent nodules, abscesses and draining tunnels with a predilection to intertriginous skin. The pathophysiology of HS is complex. However, it is known that inflammation and hyperkeratinization at the hair follicle play crucial roles in disease manifestation. Genetic and environmental factors are considered the main drivers of these two pathophysiological processes. Despite a considerable proportion of patients having a positive family history of disease, only a minority of patients suffering from HS have been found to harbor monogenic variants which segregate to affected kindreds. Most of these variants are in the *ɣ* secretase complex (GSC) protein-coding genes. In this manuscript, we set out to characterize the burden of missense pathogenic variants in healthy reference population using large scale genomic dataset thereby providing a standard for comparing genomic variation in GSC protein-coding genes in the HS patient cohort.

## Introduction

Hidradenitis suppurativa (HS) is chronic inflammatory disease of the pilosebaceous unit (PSU) characterized by recurrent nodules, abscesses and draining tunnels. These lesions can manifest in any part of the body but have a predilection to intertriginous zones such as the axillae and groin. The primary pathogenic mechanisms driving HS are inflammation and hyperkeratinization (Zouboulis et al., 2020). Several environmental and lifestyle factors play a role in disease development and progression, with obesity and smoking having strong, well-established associations with the disease (König et al., 1999; Kromann et al., 2014). In addition, HS has an underlying genetic association, with significant narrow-sense heritability estimates (77%) being reported in the literature and common forms of HS demonstrating strong familial segregation (Straalen et al., 2020). Despite several reports implicating ɣ secretase complex (GSC) variants in HS, evidence for direct causal mechanisms is lacking (Gao et al., 2006; Sellheyer and Krahl, 2008; Al-Ali et al., 2010; Miskinyte et al., 2012; Jiao et al., 2013; Nomura et al., 2014; Zhang and Sisodia, 2015; Pink et al., 2016; Ratnamala et al., 2016). For example, *in vitro* studies of variants presumed to be pathogenic, have been demonstrated to lack functional significance in relation to disease-associated pathways (Zhang and Sisodia, 2015). Conflicting results have also been obtained with regards to differential expression of genes coding for proteins of the GSC in serum and skin (Wang et al., 2010; Zouboulis et al., 2021). Therefore, despite a putative monogenic disease driver in a subset of HS patients harboring pathogenic variants, predominantly in genes coding for the protein subunits of the ɣ secretase complex (GSC) (Pace et al., 2022), the exact nature of the genetic risk in the disease remains elusive.

The GSC is a member of the intramembrane-cleavage protease (I-CliP) family that cleaves several protein substrates (Wolfe et al., 1999; Merilahti et al., 2017). The most established pathogenic role of the GSC is in familial Alzheimer’s disease (fAD) through dysregulation of amyloid precursor protein (APP) cleavage and the generation of amyloid *ß* in neural tissues (Hur, 2022). Interestingly, studies have shown that the prevalence of AD is increased four-fold in patients having a family history of HS, although no mechanistic explanation for this observation is available (Esme et al., 2020). On the other hand, the role of GSC activity in non-neural tissues such as keratinocytes and cutaneous fibroblasts is less clear. A putative mechanism implicates dysregulation of NOTCH pathway signaling in the pathophysiology of HS (Melnik and Plewig, 2013). However conflicting results have been obtained in translational studies (Frew, 2019; Frew and Navrazhina, 2020). Importantly, the molecular etiology of HS and fAD do not directly overlap. Of the GSC protein-coding genes, monogenic HS is mostly related to pathogenic variation in *NCSTN* (OMIM: #142690, Acne inversa, Familial 1), unlike fAD which is predominantly associated with *PSEN* mutations (Xiao et al., 2021; Pace et al., 2022). Additional loci involved in HS include *PSTPIP1*, *MEFV*, *NLRP3*, *IL1RN*, *NOD2* and *POFUT*, although their relevance to disease has been disputed. Despite the high proportion of cases with a family history of HS, a low prevalence of pathogenic GSC variation has been identified in sizeable patient cohorts with sporadic or familial forms of HS (Duchatelet et al., 2020). In addition, the role of common variants and polygenic risk scores in HS has not yet been evaluated.

Although the pathophysiological relevance of the GSC in humans has been extensively described, especially in Alzheimer’s disease (Hur, 2022), the variety and molecular impact of coding variants in a healthy reference population has not been evaluated. In this manuscript we use a large-scale population genomic dataset derived from the Genome Aggregation Database (GnomAD) to map the frequency and spectrum of missense variants in the four genes (*NCSTN, PSEN1, PSENEN, APH1A*) encoding protein subunits of the GSC. Evolutionary conservation metrics and *in-silico* analysis of pathogenicity are assessed, and the impact of missense variants from GnomAD on protein stability and structure and inter-subunit interactions evaluated. Thus, we provide a concise summary of the burden of GSC coding variation and its impact on protein structure in a large dataset that is routinely employed as a reference population in clinical genetic studies. Importantly, rare coding variants having deleterious *in silico* predictor scores are classified as variants of uncertain significance in the absence of definitive proof of pathogenicity, and thus they often confound molecular diagnosis. These data thereby address an important, but yet unmet, area of research which can be utilized to facilitate the interpretation of GSC missense variation in the context of associated conditions such as HS. This allows for a discussion on the implications of the findings in endotyping for precision medicine.

## Materials and methods

### Variant identification

Genomic variation in the four loci of the GSC were extracted from GnomAD v2.1.1, a large publicly-available collection of population variation from harmonized sequence data (https://gnomad.broadinstitute.org/) (Karczewski et al., 2020). The database contains a collection of putative loss of function variants (pLoF) from 125,748 exomes (containing 17.2 million variants) and 15,708 genomes (containing 229.9 million variants) from 141,456 individuals. Of these, 443,769 are high confidence pLoF variants, the majority of which involve canonical transcripts of 16,694 genes. Genomic positions are described with reference to the GRCh37/hg19 reference sequence. GnomAD contains data representative of major global European, Asian, African, and African/American ethnicities, along with additional ‘other’ populations that do not cluster with these major ethnicities. The GnomAD v2 cohort participants span a broad age range and the database does not include duplicate individuals, first or second-degree relatives to minimize inflation of rare variants. We excluded variants in GnomAD having ClinVar entries to obtain a better representation of unaffected reference populations and limit inclusion of variants reported in patient samples. pLoF variants, in-frame insertion/deletions, synonymous variants and intronic/untranslated region variants were excluded. We thus selected to focus on missense variants due to their direct clinical relevance in view of relative abundance and diversity in the human genome as well as their capacity to alter structure-function relationships at the protein level.

The observed/expected (o/e) ratio is defined as a continuous measure of how tolerant a gene is to a class of variation. A low o/e value indicates stronger evolutionary selection for that class of variation than a gene with a higher value. The o/e ratio for missense variants was compared across the four genes of the GSC to evaluate evolutionary gene constraint. The observed and expected counts depend on both gene and sample size. Thus, the 90% confidence interval for the o/e value is vital for interpretation of this metric and is presented as the 90% upper bound of the CI (termed loss-of-function observed/expected upper bound fraction, LOEUF) (Karczewski et al., 2020).

### Variant deleteriousness and conservation scoring metrics

The deleteriousness of filtered missense variants was analyzed using three *in silico* tools - Combined Annotation Dependent Deletion (CADD), PrimateAI and MetaSVM. CADD (https://cadd.gs.washington.edu/) integrates multiple annotations into a single metric (C-Score) by contrasting variants that survived natural mutation with simulated variants (Kircher et al., 2014). CADD prioritizes functional, deleterious and pathogenic variants. The resultant C-score correlates with allelic diversity and variant pathogenicity. These scores are transformed into a PHRED-like rank score relative to the genome wide distribution of scores across all potential single nucleotide variants in the human genome (8.6 × 10^9^ variants) (Rentzsch et al., 2019). A scaled CADD score of ≥20 indicates that a variant is among the top 1% most deleterious genomic variation. PrimateAI is a trained deep residual network, which interprets amino acid sequence flaking a variant of interest and the orthologous sequence alignment in other species to classify the likelihood of pathogenicity of human variants having uncertain significance (Sundaram et al., 2018). The PrimateAI score ranges from 0 to 1, with variants having a score closer to 1 being more likely to be pathogenic. MetaSVM(Liu et al., 2016) is an ensemble score using Support Vector Machine (SVM) that integrates nine prediction scores (SIFT, PolyPhen-2, GERP++, MutationTaster, Mutation Assessor, FATHMM, LRT, SiPhy and PhyloP) and allele frequencies in the 1,000 genome project (1KGP) database. The tool has been trained on 36,192 single nucleotide variants (SNVs) from UniProt.

Conservation scores (Rate4Site) were calculated using the ConSurf webserver tool (https://consurf.tau.ac.il/). This server enables estimation of the evolutionary conservation of amino/nucleic acid positions in a macromolecule based on the phylogenetic relations between homologous sequences (Ashkenazy et al., 2016). The degree of conservation at a specific residue correlates to its structural and functional relevance. ConSurf provides accurate computation of evolutionary rate by using an empirical Bayesian method. The tool normalizes scores so that the average score for all residues is zero whilst the standard deviation is one. Negative values represent slowly evolving, highly conserved sites with the most conserved position having a score of −1.

### The effects of missense variants on protein stability and inter-subunit interaction

The filtered missense variants were cross-referenced to Missense3D-DB, a database of precomputed structural predictions for approximately 4 million human missense variants (Khanna et al., 2021). Missense3D enables evaluation of the impact of missense variants on protein structure. It assesses 17 different structural features that are essential for protein confirmation and stability, such as stearic clashes and disallowed phi/psi angles, to identify substitutions that are conformationally deleterious (Venselaar et al., 2010). VarSeq software (Golden Helix) was used for variant interpretation and annotation (Kopanos et al., 2019).

Protein structures as predicted by AlphaFold were utilized using the following identifiers; AF-Q96BI3-F1 (APH1A), AF-Q92542-F1 (Nicastrin), AF-P49768-F1 (PSEN1) and AF-Q9NZ42-F1 (PSENEN). Structural representations were generated using the PyMOL molecular visualization system (PyMOL, 2020). Protein domains were identified using InterPro Database14 and drawn using ProSite online software (https://prosite.expasy.org/). Protein domains were related to the distribution of evolutionary conservation and deleteriousness across primary structure as outlined above.

The estimated stability spectrum of all identified missense variants in GSC was evaluated using FoldX 5.1 (Schymkowitz et al., 2005). To determine how a single point substitution affects protein stability, ΔΔG (kcal/mol) was calculated. This metric quantifies the difference in the change of Gibbs-free energy and is a measure of the change in energy between the folded and unfolded states (ΔG_folding_) and the change in ΔG_folding_ when a missense variant is present. ΔΔG is thus a predictor of whether a missense variant will be energetically favorable in terms of protein stability. Using FoldX 5.1, the energies for the wild type (ΔG_fold,WT_) and the mutant (ΔG_fold,MT_) protein were computed to give the stability change (ΔΔG_fold_ = ΔG_fold,MT_ − ΔG_fold,WT_).

The change in Gibbs-free energy (ΔΔG_folding_ in kcal/mol) was categorized based on the following thresholds: highly stabilizing (≤−1.84), stabilizing (−1.84 to −0.92), slightly stabilizing (−0.92 to −0.46), neutral (−0.46 to +0.46), slightly destabilizing (+0.46 to +0.92), destabilizing (+0.92 to +1.84), and highly destabilizing (>+1.84) (Studer et al., 2014).

A systematic *in-silico* evaluation of residues at inter-subunit protein-protein interactions (PPI) in the GSC was performed using BUDE Alanine Scan (https://pragmaticproteindesign.bio.ed.ac.uk/balas/) (Wood et al., 2020). This tool enables characterization of the thermodynamic stability of PPI hotspot residues using computational alanine scanning. *In-silico* alanine scanning mutagenesis identifies residues which contribute most to the change in the free energy of binding ΔΔG (referred to a “hot spots” (Clackson and Wells, 1995)) defined as ΔΔG = ΔG^mut^–ΔG^wt^ where ΔG^wt^ refers to the binding free energy upon complexing of the wild-type residue and ΔG^mut^ to that with alanine-mutated proteins. Neutral residues are defined as residues which when mutated fail to meet the ΔΔG ≥2.0 kcal/mol hot spot residue criterion (Morrow and Zhang, 2012).

## Results

### The spectrum of missense variants in *ɣ* secretase complex protein-coding genes

**FIGURE 1:**
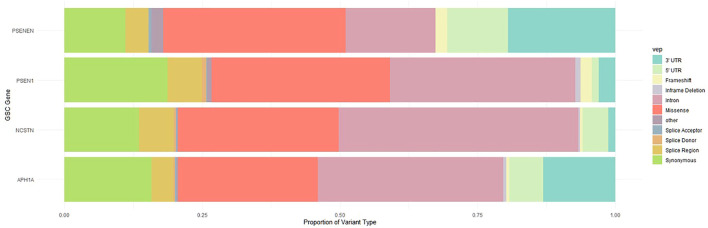
Proportion of variant types across these four GSC protein-coding genes.

Using aggregate, multi-ancestry whole exome and whole genome sequencing data from GnomAD, a broad spectrum of variants in *NCSTN*, *PSEN1*, *PSENEN* and *APH1A* were identified. The proportion of variant types across these four GSC protein-coding genes is illustrated in Figure 1. As expected, deleterious variants that disrupt translation through nonsense, frameshift or splice-altering effects are maintained at low frequencies in human populations, whereas missense and synonymous variants are more abundant. A total of 524 missense variants at these four loci were filtered from GnomAD, with most variants (*n* = 300) located in *NCSTN,* the largest of the four genes, followed by 131, 48, 45 missense variants in *PSEN1*, *APH1A* and *PSENEN* respectively. The global observed/expected (o/e) evolutionary constraint score for missense variants in the GSC genes varies, with an o/e ratio of 0.79 (0.72–0.87) for *NCSTN*, 0.62 (0.55–0.71) for *PSEN1*, 0.75 (0.59–0.98) for *PSENEN* and 0.54 (0.46–0.65) for *APH1A.* This indicates that *APH1A* is less tolerant to missense variation compared to the other GSC loci. In comparison, the four loci have a high probability of being loss-of-function intolerant (pLI) with the loss-of-function o/e upper bound fraction as described by GnomAD being lower than 0.5 (Karczewski et al., 2020).

**FIGURE 2:**
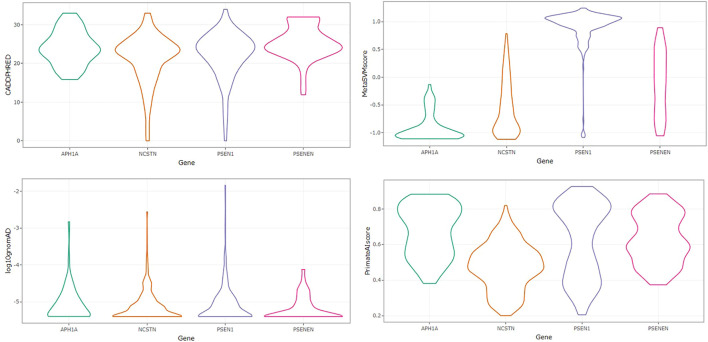
Violin plots showing the distribution of PHRED-scaled CADD scores, MetaSVM, variant allele frequencies, and PrimateAI scores across *APH1A, NCSTN, PSEN1* and *PSENEN.*

The distribution of variant allele frequencies across the four loci is shown in Figure 2 with a higher median variant allele frequency in *APH1A* and *NCSTN*. Importantly, the four loci are characterized by abundant rare variants and a relative paucity of missense variants exceeding 0.1% in minor allele frequency. Most of the identified missense variants are singletons carried by only a single individual in GnomAD. This corresponds to the negatively skewed distribution of log_10_ allele frequency around -5.5 in the four genes. We also compared the distribution of CADD and PrimateAI scores across the four GSC loci. Significantly higher CADD scores occur in *PSENEN*, *PSEN1* and *APH1A* compared to *NCSTN* (Kruskal–Wallis ANOVA, *p* < 0.05). PrimateAI scores were also significantly higher in *PSEN1*, *APH1A* and *PSENEN* relative to *NCSTN* (Kruskal–Wallis ANOVA, *p* < 0.05). The CADD and PrimateAI scores correlate well (r_s_ = 0.627, *p* < 0.001) and both functional prediction algorithms thus illustrate an identical pattern of deleteriousness across the four genes. The distribution of MetaSVM scores was significantly higher in *PSEN1* compared to the other three loci (Kruskal–Wallis ANOVA, *p* < 0.05) as shown in Figure 2.

**FIGURE 3:**
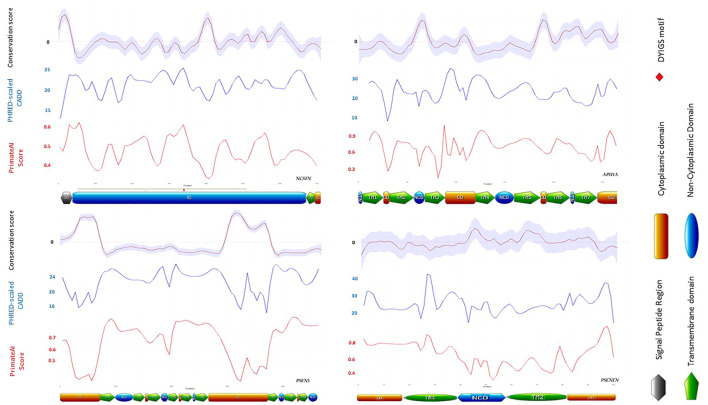
Distribution of CADD, PrimateAI and Rate4S scores across protein domains of the four GSC subunits. The nicastrin DYIGS motif illustrated by a red marker is highly conserved, and variation in this region is highly deleterious.

Given these findings, we set out to map the conservation and pathogenicity scores of missense variants across the primary amino acid sequence of each of the four GSC genes. As expected, a clear interrelatedness of conservation and deleteriousness is demonstrated, with the most conserved residues being also the most deleterious based on Phred-CADD and PrimateAI scores as illustrated in Figure 3. We also evaluated the distribution of CADD, PrimateAI and Rate4S scores across protein domains. A higher distribution of CADD scores was observed in the peptidase domain of nicastrin (Residues 254–468) compared to the other regions, although this did not exceed statistical significance thresholds. Notably, a high pathogenicity and conservation score was observed in the DYIGS motif located in a proposed nicastrin substrate-binding pocket (Xie et al., 2014). The four loci are characterized by a high burden of putatively deleterious variation, with most positions having CADD scores >20.

### Impact on *ɣ* secretase complex protein structure

**FIGURE 4:**
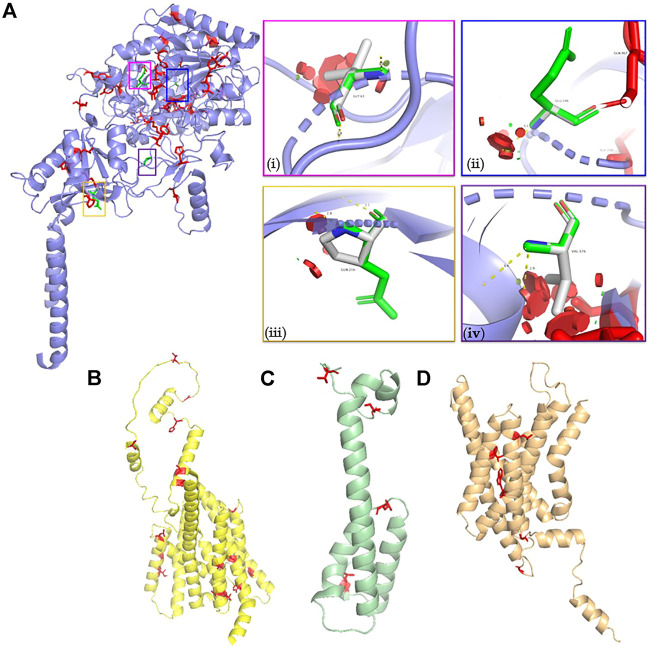
Molecular models of **(A)** NCSTN **(B)** PSEN **(C)** PSENEN and **(D)** APH1A proteins. Structurally deleterious residues are highlighted as red ball and stick residues. The structurally damaging *NCSTN* missense variants described in HS are illustrated in vignettes (i) p. Gly61Val (ii) p. Glu296Gly (iii) p. Gln216Pro, and (iv) p. Gly576Val. The p. Gly61Val triggers a disallowed phi/psi angle and replaces a buried glycine residue. The p. Gly296Gly variant also triggers a disallowed phi/psi angle. Finally, the p. Gly296Gly variant replaces a burred charged residue with an uncharged residue. It also leads to expansion of the cavity volume by 139.9 Å3.

The burden of structurally deleterious missense variants in the GSC was evaluated using the Missense3D server as outlined in the methods. Approximately 13% of all the reported GnomAD coding variants in *NCSTN*, *APH1A* and *PSEN1* were predicted to be structurally deleterious. Conversely, a lower proportion (8%, 4/45) of variants in *PSENEN* were structurally deleterious. GSC variants with a structurally deleterious impact on protein structure are highlighted red in ball and stick mode in Figure 4. In comparison, four (namely p. Gly61Val (Duchatelet et al., 2020), p. Gln216Pro (Zhang et al., 2013), p. Glu296Gly (Xu et al., 2016) and p. Gly576Val (Duchatelet et al., 2020) [residues in ball and sick mode highlighted in green]) of the ten *NCSTN* missense variants reported in HS are deleterious (40%, *χ* ^2^ = 6.01, *p* < 0.05) (Pace et al., 2022). The criteria for deleteriousness is illustrated in Supplementary Table S1. This indicates significant enrichment for damaging missense *NCSTN* substitutions in HS. The normalized conservation score according to ConSurf for the wild type residues at these four positions are −0.885, −0.888, −0.540 and −0.889 respectively, indicating that these variants are slowly evolving, highly conserved sites. Conversely, none of the reported *PSEN* and *PSENEN* missense variants in HS are structurally deleterious. No disease associated *APH1A* missense variant has been reported to date, although a single missense variant in its homolog *APH1B* has been published in association with the HS phenotype (Theut Riis et al., 2020). This *APH1B* p. His170Arg substitution is structurally damaging; however, the authors had queried the relevance of this gene in HS, since functional models demonstrate a higher cutaneous expression of *APH1A* rather than *APH1B* (Theut Riis et al., 2020).

### The energy stability landscape of *ɣ* secretase coding variants

**FIGURE 5:**
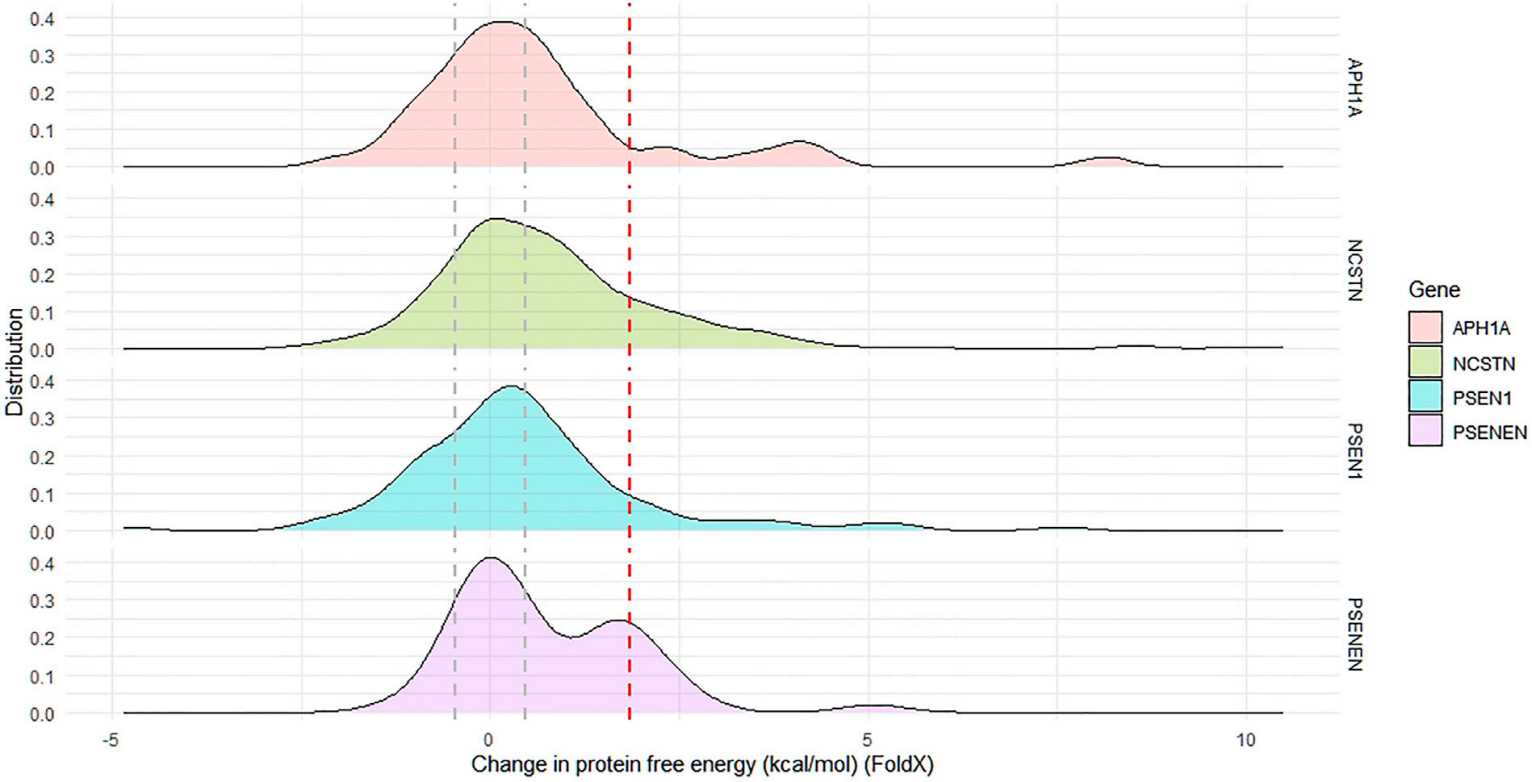
The distribution of change in protein free energy in proteins forming the gamma secretase complex. The global distribution of APH1A and PSENEN in bimodal. The area between the grey dotted lines indicate proportion of reported missense variants with net-neutral change in free energy, whilst the region beyond the dotted red line indicates the proportion of missense variants having a highly destabilizing change in free energy.

The effect of all GnomAD-reported missense variants in the GSC on protein stability was investigated using FoldX as outlined in the methods. This analysis identifies positions that are mutation intolerant. Across the GSC, 46% of variants are destabilizing according to ΔΔG calculations. The distribution curves and ΔΔG cut-offs for each locus are shown in Figure 5. The global ΔΔG distribution for *APH1A* is also bimodal, with the top destabilizing variants in this gene being p. Ser213Arg, p. Trp174Arg and p. Tyr90Cys. Similarly, the ΔΔG distribution for *PSENEN* is bimodal, with the most destabilizing variants in this gene being p. Leu26Pro and p. Ser94Phe. On the other hand the global distribution of ΔΔG for all *NCSTN* and *PSEN1* variants in GnomAD is unimodal and skewed towards positive values. For the *NCSTN* polymorphisms, the topmost thermodynamically destabilizing variants with extreme ΔΔG values (>6.0 kcal/mol) are p. Val96Phe, p. Leu282Pro, p. Ser286Phe and p. Asp381His. For *PSEN1*, the topmost thermodynamically destabilizing variants are p. Gly266Val, p. Leu364Pro, p. Ser367Arg and p. Ser313Pro. Across the four GSC loci, a significantly higher ΔΔG value was present in substitutions that had deleterious structural predictions by Missense3D.

### *In-silico* alanine scanning identifies *ɣ* secretase hotspot residues at protein-protein interfaces

Protein-protein interactions are dependent on biochemical and/or electrostatic forces and are an essential element of the function of multi subunit complexes. Given their significant role in biological function, disruptions at interface residues can lead to disease. Five of the filtered GSC missense variants which are predicted to be structurally damaging by Missense3D are located at the protein’s interface as per data from the RCSB’s protein data bank (rcsb.org) (Berman et al., 2000). These include: *PSEN1*:p.Leu219Phe, *APH1A:*p.Gly233Arg and *PSENEN*:p.Leu3Gln, p. Leu26Pro and p. Gly99Asp. *In silico* alanine scanning using BudeAlaScan (Wood et al., 2020) identified hotspots which are presented in Supplementary Table S2. Of the filtered missense variants across the four GSC genes predicted to be structurally deleterious, only the *PSENEN:*p.Leu26Pro variant disrupts a hot spot residue.

On the other hand, of the pathogenic/likely pathogenic variants described in HS patients with multigenerational disease, only the *NCSTN*:p.Val224_Thr227del variant spans a hotspot residue. The deleted residues in this variant are located within an α-helix of NCSTN. Hydrophobic interactions between nicastrin residues Val224 and Ile225 and PSENEN’s Pro101 are disrupted by this variant. Therefore, it is possible that the in-frame deletion p. Val224_Thr227 in *NCSTN* influences both the structure and the stability of the *NCSTN* ectodomain and sterically hinders GSC-substrate interactions (Mintoff et al., 2021).

## Discussion

In this study we describe an *in-silico* evaluation of coding variation in the GSC, derived from the GnomAD reference population dataset. Several attributes of missense variation in *NCSTN*, *PSEN1*, *PSENEN* and *APH1A* are assessed, including evolutionary conservation and deleteriousness metrics and their relation to protein domains. The impact of amino acid substitutions on protein structure, thermodynamic stability and inter-subunit interaction hotspots is also evaluated. Genes of the GSC have an established role in the pathogenesis of both monogenic Alzheimer disease (primarily *PSEN1*) (Sims et al., 2020) and familial HS (primarily *NCSTN*), although genetic studies of HS are still in their infancy and characterized by relatively small numbers. In addition, variation within the GSC has been associated with complex non-Mendelian traits, ranging from educational attainment to forced expiratory volume in chronic obstructive pulmonary disease to apolipoprotein B levels (Lutz et al., 2015; Lee et al., 2018; Richardson et al., 2020). To the best of our knowledge, this study provides the first comprehensive characterization of GSC variation in a large population genomic dataset, and its interpretation in the setting of known HS pathogenic variation to date.

The analysis demonstrates a high burden of deleterious variation in the four GSC loci as determined by CADD and MetaSVM metrics, and a clear correlation between putative deleteriousness and evolutionary selection pressure along the primary structure of each protein. We also demonstrate significant enrichment for structurally damaging *NCSTN* missense variants in HS relative to GnomAD, with the affected residues being under strong selection pressure. *PSEN* and *PSENEN* missense variants reported in HS phenotypes are not structurally deleterious, suggesting that these can contribute to disease via different mechanisms. Energy calculations reveal that several substitutions are likely to cause a thermodynamically unfavorable effect, and analysis of protein-protein interfaces identifies hotspot residues where substitution is less energetically favorable and likely to cause destabilization of the complex.

The results of this analysis suggest that residues having low Rate4Site scores, high CADD and PrimateAI scores, high ΔΔG energies and structurally deleterious effects based on Missense3D should be prioritized for possible functional analysis to determine their impact on GSC function. While evolutionary conservation metrics provide valuable insight, it is important to consider that their substitution does not always result in disease. It has been postulated that the observed conservation is the result of reduced fitness that the nowadays-benign variants could elicit in affected hosts when competing for survival in the wild (Zaucha et al., 2020). Thus, conservation metrics should not be interpreted in isolation.

Although HS is a heritable trait (Bruinsma et al., 2021), only a minority of patients with apparent familial disease have underlying GSC pathogenic variation. Data from twin registries suggests that the primary mechanism underlying HS involves gene-gene and gene-environment interactions rather than monogenic variation (Straalen et al., 2020; Kjaersgaard Andersen et al., 2021). The extent and mechanisms by which variation in the GSC is implicated in the development and progression of HS is as yet to be elucidated. Indirect evidence in support of GSC involvement in the pathophysiology of HS includes the induction of disease in 75% of patients administered Nirogacestat, a selective pharmacological *ɣ*-secretase inhibitor (O’Sullivan Coyne et al., 2018). On the other hand, functional studies reveal that variation in GSC subunits alone (specifically *PSENEN*) may not suffice for the development of HS (Zhou et al., 2021).

Whole exome or whole genome sequencing remain the primary methodological approaches of choice for the genetic investigation of patients with suspected familial or syndromic HS (Rehm et al., 2013). Both techniques enable implementation of a biologically-agnostic, hypothesis-free approach. Exome sequencing has been shown to enable diagnoses of 20% of otherwise undiagnosed rare conditions (Gahl et al., 2012). One of the major drawbacks of expansive high-throughput sequencing is the detection of novel or rare missense variations of uncertain significance (VUSs). Such VUS are characterized by the absence of robust evidence to support relevance to a given clinical phenotype yet simultaneously possessing properties reminiscent of disease-causing variation. VUSs are frequent and thus pose a major clinical challenge (Cooper, 2015; Richards et al., 2015; Federici and Soddu, 2020). Rare or novel missense variants often remain relegated to the VUS category thus restricting their interpretation, clinical actionability and dissemination. As a consequence, VUSs place a burden on decision-making and communication. Importantly, population allele frequencies from GnomAD exomes and genomes are routinely employed by variant pathogenicity classification algorithms (Kopanos et al., 2019). Against this background, the present study gains significance as it characterizes GnomAD coding variants which create structural or functional alterations in the GSC.

Phenotypic heterogeneity adds to the complexity of HS, since the conditions presents with extensive variation in affected corporeal sites, underlying risk factors, clinical presentation, disease course and response to therapy. However, this can be considered an avenue for characterizing disease pathophysiology, as it also implies heterogenous pathogenic pathways in HS (Frew et al., 2015). This is reflected in patient endotype clustering. A proposed HS patient cluster having underlying GSC variation, was described as presenting with early-onset disease and a lean constitution, being more likely to have lesions in posterior sites, nodular disease, and suffer from co-morbid pilonidal sinus disease (González-Manso et al., 2021). On the other hand, only weak genotype-phenotype associations have been made. Patients harboring *NCSTN* variants have been described as being likelier to exhibit a phenotype characterized by axillary and mammary involvement, hypertrophic scars, more likely to be females and less likely to be smokers (Axillary-Mammary, LC1 phenotype (Canoui-Poitrine et al., 2013)) (Frew et al., 2019). Interestingly, this patient group is less likely to report a family history of disease. Such association is likely to be confounded by the fact that most HS patients are females having disease involving the axilla and/or breast (Dufour et al., 2014). Appropriate classification has been described as “essential” for providing personalized medicine for HS patients (Ingram and Piguet, 2013). It may also help to reduce the diagnostic delay which invariably leads to a worse prognosis (Kokolakis et al., 2020). Further inferred evidence for phenotypic and pathophysiological heterogeneity may be gained from investigating the genetic underpinnings of HS. The GSC, particularly nicastrin, may contribute more towards inflammation at PSU (Frew and Navrazhina, 2019; Hermasch et al., 2022), possibly through NOTCH pathways,. Other as yet unidentified genes may contribute more toward hyperkeratinization of the PSU’s infundibular segment.

Genomic studies in HS and the use of GnomAD in variant classification are characterized by several caveats that we will outline next. A comprehensive understanding of the impact of GSC missense variants on protein function is lacking. Attributing pathogenicity is difficult in the absence of *in-vitro* or *in-vivo* functional evidence. Few studies investigate changes in substrate binding affinity, NOTCH reporter assays or catalytic activity of the GSC, and thus follow-up of rare variants identified from sequencing studies is required. The spectrum of locus heterogeneity driving HS is unknown. Several independent reports have implicated *NCSTN* variation in lean subjects with atypical or familial HS. Yet, it is important to consider that selecting for such probands or kindreds can lead to an ascertainment bias that overestimates the contribution of the GSC in HS. It is thus reasonable to postulate that variants having reduced penetrance which predispose to milder or late-onset disease and that do not drive familial segregation are not detected in cohort studies. Such hypomorphic or low-risk variants necessitate large discovery and replication cohorts. Ranola *et al.* show that comparing clinical cases to population controls derived from public databases ascertained using different criteria, can introduce false positives in genetic testing (Ranola et al., 2019). Important lessons can be derived from studies on transethnic comparison of polygenic risk scores. These highlight the need for an understanding of population-specific genetic risk and reinforce the rationale for large-scale genome-wide association studies in diverse populations (Duncan et al., 2019; Matsunaga et al., 2020).

In comparison to other monogenic diseases, the use of GnomAD-derived allele frequencies in HS carries some distinctions. Deriving allele frequencies from aggregate datasets unselected for disease, such as GnomAD, is a powerful approach that is core in ACMG-AMP classification criteria. However, rare variants causing late-onset disease are present in GnomAD. These can confound variant classification, and the causality of previously identified disease-associated variants has been disputed (Manrai et al., 2016; Kosmicki et al., 2017). For low-frequency missense variants that are not structurally deleterious and present in GnomAD, attributing causality requires functional evaluation. Conversely, it is impossible to ascertain whether diseases such as HS are represented in GnomAD, since phenotype and other individual-level data is not included. GnomAD excluded participants with severe pediatric diseases that impact on reproductive fitness. As HS is characterized by a significant diagnostic delay and extensive clinical heterogeneity in adult patients, it is likely that GnomAD participants bear HS-causal GSC variants. This reinforces the need for large biobank-driven prospective genomics HS research endeavors with carefully phenotyped cohorts.

In addition to the restrictions imposed by the absence of individual-level phenotype data in GnomAD, additional limitations must be acknowledged. GnomAD contains an overrepresentation of European participants, with poor representation from Asian, Oceanic and Middle Eastern populations. No knowledge on the penetrance of GSC alleles in HS exists. We focused on missense variants only. However, variants in introns, promoter regions or synonymous substitutions can impact on transcriptional efficiency or alter splicing. We did not stratify the analysis based on population substructure or gender data within GnomAD. Additionally, the alternate effect of substitutions on splicing isoforms that might offset the deleterious effects of variants was not considered (Kukurba et al., 2014; Miosge et al., 2015). The use of protein 3D structural data to computationally model the effects of missense variants can provide additional evidence for deleteriousness. Methods that assess the thermodynamic properties related to mutations, such as changes in protein stability consider only one possible mechanism that may affect the phenotype (Glusman et al., 2017). It is likely that missense variants produce functional changes through alternate molecular mechanisms. *In-silico* pathogenicity classifiers were based on output from MetaSVM and CADD. Recent research suggests that gene-specific thresholds provide better performance for missense variants in a subset of genes (Sallah et al., 2022). MetaSVM has been shown to yield high performance in benchmark studies (Anderson and Lassmann, 2018) although, some investigations showed that it can exaggerate pathogenicity of variants in specific proteins (Zaucha et al., 2020). The overreliance on *in-silico* predictors in the absence of their validation in the setting of HS-causal GSC variation is another limitation. This study only made use of the GnomAD dataset, and did not consider additional sources of reference genomic data such as the United Kingdom Biobank and TopMed repositories (Sudlow et al., 2015; Taliun et al., 2021).

## Conclusion

This study highlights the need for a greater effort to be made in the understanding of the sequence-function relationship of the GSC in HS. Importantly, a combined clinical and molecular characterization approach is required to capture subtle pathophysiological changes along the disease continuum involving both hyperkeratinization and inflammation. How alleles in GSC act to modify susceptibility in the context of a multifactorial trait, including interaction with complex polygenic risk phenotypes such as obesity or the metabolic syndrome, is also unknown. We envisage that the future of identifying and validating novel disease-gene candidates, may occur through interdisciplinary collaboration of clinical and research facilities (Rehm et al., 2013). Various hurdles need to be overcome for HS genomics research to achieve clinical significance, including deep phenotyping, screening of at-risk family members to delineate segregation, and detailed variant curation (Jabbour et al., 2021). Rare-variant burden enrichment analysis provides additional scope for HS and has recently been applied to GSC-associated syndromic forms of HS (Brandão et al., 2022). This study also highlights the necessity to harness insights from structural biology into clinical variant interpretation algorithms.

## Data Availability

The datasets presented in this study can be found in online repositories. The names of the repository/repositories and accession number(s) can be found in the article/Supplementary Material.
