# Supplementary material for: Interpreting the spectrum of gamma-secretase complex missense variation in the context of hidradenitis suppurativa—An in-silico study
Source: Front Genet. 2022 Sep 2;13:962449. doi: 10.3389/fgene.2022.962449 (PMC9478468; doi:10.3389/fgene.2022.962449)
Supplement: Supplementary file 1 [file DataSheet1.docx]

**Table S1:** The criteria for deleteriousness of the *NCSTN* missense variants described in patients with Hidradenitis Suppurativa

| ***NCSTN* Variant** | **Structural features of mutation site** | **Missense3D** | **Structure –based stability predictions** |
| --- | --- | --- | --- |
| **p.Gly61Val** | The substitution replaces a buried glycine with a larger, hydrophobic residue and may impact on the flexibility provided by wild type glycine. The wild type residue is not fully conserved. | The substitution is structurally damaging. It triggers a disallowed phi/psi alert. The phi/psi angles are in favoured region for wild-type residue but outlier region for mutation residue. The substitution also replaces a buried GLY residue (RSA2.3%) with a buried VAL residue (RSA2.8%) | ΔΔG mCSM: Destabilizing (-0.353kcal/mol)  ΔΔG SDM: Destabilizing (-.440 kcal/mol)  ΔΔG DUET: Destabilizing (-0.037 kcal/mol)  ΔΔS_Vib_ ENCoM: -0.781 kcal.mol^-1^.K^-1^ (Decrease of molecule flexibility) |
| **p.Gln216Pro** | The variant residue is smaller than the wild type residue. Them variant residue is more hydrophobic than the wild-type residue. This can result in loss of hydrogen bonds and/or disturb correct folding. The wild type residue is predicted to be located in an a-helix. Proline disrupts an a-helix when not located at one of the first 3 positions of that helix. In case of the variant at hand the helix will be disturbed and this can have effects on the structure of the protein. | This substitution triggers disallowed phi/psi alert. The phi/psi angles are in favoured region for wild-type residue but outlier region for variant residue* | ΔΔG mCSM: -0.388 kcal/mol (Destabilizing) ΔΔG SDM: -1.810 kcal/mol (Destabilizing) ΔΔG DUET: -0.637 kcal/mol (Destabilizing)  ΔΔS_Vib_ ENCoM: -0.021 kcal.mol^-1^.K^-1^ (Decrease of molecule flexibility) |
| **p.Glu296Gly** | The substitution replaces a negatively charged residue with a smaller, more flexible, hydrophobic, neutral residue | The substitution is structurally damaging. It replaces a buried charged residue (Glu, RSA 0.0%) with an uncharged residue (Gly). It also leads to the expansion of the cavity volume by 139.9 Å^3^ | ΔΔG mCSM: Destabilizing (-0.380kcal/mol)  ΔΔG SDM: Destabilizing (-1.000 kcal/mol)  ΔΔG DUET: Destabilizing (-0.523 kcal/mol  ΔΔS_Vib_ ENCoM: 1.029 kcal.mol^-1^.K^-1^ (Increase of molecule flexibility) |
| **p.Gly576Val** | The variant residue is bigger, more flexible and more hydrophobic than the wild type residue | The substitution is structurally damaging as it replaces a glycine located in a bend curvature. | ΔΔG mCSM: Destabilizing (-0.625kcal/mol)  ΔΔG SDM: Destabilizing (-0.970 kcal/mol)  ΔΔG DUET: Destabilizing (-0.628 kcal/mol)  ΔΔS_Vib_ ENCoM: -1.420 kcal.mol^-1^.K^-1^ (Decrease of molecule flexibility) |

**Table S2:** Hotspot residues at interface ɣ secretase proteins identified by *in silico* alanine scanning

| **Chain** | **Residue** | **Amino Acid** | **ΔΔG (kJ/mol)** |
| --- | --- | --- | --- |
| APH1 | 5 | VAL | 2.2431 |
| APH1 | 7 | PHE | 2.3003 |
| APH1 | 11 | PHE | 11.1073 |
| APH1 | 12 | VAL | 3.1454 |
| APH1 | 16 | PRO | 2.0585 |
| APH1 | 18 | PHE | 2.7584 |
| APH1 | 20 | LEU | 2.0061 |
| APH1 | 23 | ILE | 7.9623 |
| APH1 | 29 | PRO | 2.838 |
| APH1 | 32 | VAL | 2.1535 |
| APH1 | 33 | ILE | 6.0687 |
| APH1 | 36 | VAL | 4.6026 |
| APH1 | 40 | PHE | 11.7636 |
| APH1 | 42 | TRP | 4.7704 |
| APH1 | 43 | LEU | 7.9115 |
| APH1 | 46 | LEU | 6.615 |
| APH1 | 47 | LEU | 6.9488 |
| APH1 | 50 | SER | 3.5723 |
| APH1 | 53 | TRP | 14.9408 |
| APH1 | 54 | PHE | 5.9504 |

| NCSTN | 36 | VAL | 6.6807 |
| --- | --- | --- | --- |
| NCSTN | 39 | LYS | 12.7785 |
| NCSTN | 40 | ILE | 8.3871 |
| NCSTN | 41 | TYR | 17.8798 |
| NCSTN | 42 | ILE | 2.1895 |
| NCSTN | 63 | GLN | 2.672 |
| NCSTN | 220 | HIS | 2.8473 |
| **NCSTN p.Val224_Thr227del** | **224** | **VAL** | **3.192** |
| NCSTN | 232 | ARG | 3.328 |
| NCSTN | 233 | ARG | 12.2876 |
| NCSTN | 236 | ILE | 5.3851 |
| NCSTN | 237 | GLN | 5.3893 |
| NCSTN | 240 | PHE | 4.6895 |
| NCSTN | 241 | SER | 2.8721 |
| NCSTN | 242 | ILE | 2.5227 |
| NCSTN | 243 | ASN | 4.067 |
| NCSTN | 245 | GLU | 5.9076 |
| NCSTN | 661 | PHE | 5.5451 |
| NCSTN | 662 | LEU | 5.438 |

| PSEN1 | 83 | ILE | 4.7682 |
| --- | --- | --- | --- |
| PSEN1 | 86 | PHE | 6.3289 |
| PSEN1 | 109 | LYS | 3.4612 |
| PSEN1 | 179 | PHE | 5.1479 |
| PSEN1 | 180 | ILE | 2.1028 |
| PSEN1 | 186 | PHE | 3.3011 |
| PSEN1 | 187 | LYS | 11.9316 |
| PSEN1 | 190 | ASN | 2.7719 |
| PSEN1 | 193 | VAL | 3.4709 |
| PSEN1 | 194 | ASP | 6.1909 |
| PSEN1 | 195 | TYR | 17.0328 |
| PSEN1 | 196 | ILE | 2.9017 |
| PSEN1 | 198 | VAL | 2.0224 |
| PSEN1 | 200 | LEU | 7.049 |
| PSEN1 | 203 | TRP | 11.6593 |
| PSEN1 | 204 | ASN | 9.1514 |
| PSEN1 | 208 | VAL | 5.1617 |
| PSEN1 | 211 | ILE | 3.8923 |
| PSEN1 | 215 | TRP | 3.9073 |
| PSEN1 | 216 | LYS | 4.02 |

| PEN2 | 10 | GLU | 2.1898 |
| --- | --- | --- | --- |
| PEN2 | 14 | LEU | 2.753 |
| PEN2 | 18 | TYR | 9.7531 |
| PEN2 | 25 | PHE | 2.2214 |
| PEN2 | 26 | LEU | 8.4681 |
| PEN2 | 28 | PHE | 9.9688 |
| PEN2 | 29 | LEU | 3.4281 |
| PEN2 | 32 | VAL | 3.712 |
| PEN2 | 36 | TRP | 6.4314 |
| PEN2 | 67 | TRP | 3.1388 |
| PEN2 | 71 | LEU | 3.9144 |
| PEN2 | 74 | TRP | 6.302 |
| PEN2 | 75 | ILE | 2.6517 |
| PEN2 | 78 | PHE | 7.4644 |
| PEN2 | 79 | GLN | 3.2321 |
| PEN2 | 90 | ASP | 5.5058 |
| PEN2 | 91 | TYR | 4.9825 |
| PEN2 | 92 | LEU | 2.1632 |
| PEN2 | 94 | PHE | 21.8483 |
| PEN2 | 95 | THR | 6.3549 |
| PEN2 | 96 | ILE | 5.6812 |
| PEN2 | 97 | PRO | 2.6189 |
| PEN2 | 98 | LEU | 5.6087 |
| PEN2 | 100 | THR | 2.1619 |
